# Supplementary material for: Quantifying the burden of hereditary hemorrhagic telangiectasia on quality of life and psychological health: a cross-sectional study
Source: Orphanet J Rare Dis. 2025 Mar 7;20:109. doi: 10.1186/s13023-025-03620-8 (PMC11889918; doi:10.1186/s13023-025-03620-8)
Supplement: Supplementary file 1 — Supplementary Material 1 [file 13023_2025_3620_MOESM1_ESM.docx]

**SUPPLEMENT 1**

**Quality of Life Survey For Those Living With Hereditary Hemorrhagic Telangiectasia**
