# Supplementary material for: Quantifying the burden of hereditary hemorrhagic telangiectasia on quality of life and psychological health: a cross-sectional study
Source: Orphanet J Rare Dis. 2025 Mar 7;20:109. doi: 10.1186/s13023-025-03620-8 (PMC11889918; doi:10.1186/s13023-025-03620-8)
Supplement: Supplementary file 2 — Supplementary Material 2 [file 13023_2025_3620_MOESM2_ESM.docx]

**SUPPLEMENT 2**

eTable 1. Multivariable Linear Regression Analysis With Epistaxis Severity and Demographic Variables As Covariates of Quality of Life Outcomes.

eTable 2. Tukey’s Honestly Significant Difference (Tukey’s HSD) Test Analysis Results

eTable 3: Kruskal-Wallis Test

eTable 4: Symptoms for Which the Participant Would Like to See Improvement With Treatment (Answer: “Yes, I would like to see this symptom improved with treatment.”)

eTable 5. Five-Point Likert Scale of Importance for Patients to Improve HHT-Related Diagnoses

eTable 6. Five-Point Likert Scale of Degree of HHT Impact in Areas of Functioning

eTable 7. Five-Point Likert scale of Importance for Patients to Improve Different Areas of Functioning.

**eTable 1** Multivariable Linear Regression between epistaxis severity and demographic variables covariates (age, sex, race, and ethnicity), and HRQoL measurements (HADS, PROMIS-Fatigue 8a, and SF-36) as outcomes.

| **HADS-A** | | | **HADS-D** | | **PROMIS-8a Fatigue scale** | | **SF-36 PCS** | | **SF-36 MCS** | |
| --- | --- | --- | --- | --- | --- | --- | --- | --- | --- | --- |
| Covariate | **Beta (SE)** | **95% CI** | **Beta (SE)** | **95% CI** | **Beta (SE)** | **95% CI** | **Beta (SE)** | **95% CI** | **Beta (SE)** | **95% CI** |
| Age (years) | −0.1 (0.01) | −0.1 to −0.04 | −0.01 (0.01) | −0.03 to 0.01 | −0.07 (0.02) | −0.1 to 0 | −0.4 (0.1) | −0.5 to −0.2 | 0.2 (0.06) | 0.1-0.3 |
| Sex (female) | 0.8 (0.4) | −0.00 to 1.6 | −0.32 (0.4) | −1 to 0.4 | 1.9 (0.8) | 0.3-3.5 | −7.3 (2.7) | −12.7 to −1.8 | −0.8 (1.9) | −4.5 to 2.9 |
| Race (Other) | Ref. |  | Ref. |  | Ref. |  | Ref. |  | Ref. |  |
| White | −1.1 (0.7) | −2.59 to 0.3 | −0.48 (0.6) | −1.8 to 0.8 | 0.5 (1.5) | −2.4 to 3.4 | 11 (5) | 1-20.9 | 4.7 (3.5) | −2.1 to 11.5 |
| ESS(Mild) | Ref. |  | Ref. |  | Ref. |  | Ref. |  | Ref. |  |
| Moderate | 0.35 (0.4) | −0.4 to 1.1 | 1.15 (0.4) | 0.5-1.8 | 3.8 (0.8) | 2.3 - 5.3 | −12.1 (2.6) | −17.3 to −6.8 | −2.7 (1.9) | −6.4 to 0.9 |
| Severe | 2.4 (0.5) | 1.5-3.4 | 3.32 (0.4) | 2.5-4.2 | 8.1 (1) | 6.2 - 10 | −26.4 (2.6) | −32.9 to −19.8 | −10.7 (2.3) | −15.4 to 6 |
| NOSE-HHT(Mild) | Ref. |  | Ref. |  | Ref. |  | Ref. |  | Ref. |  |
| Moderate | 0.8 (0.4) | −0.07 to 1.6 | 1.3 (0.3) | 0.5-2 | 3.8 (0.8) | 2.2-5.3 | −6.2 (2.7) | −11.6 to −0.8 | −6.5 (2) | −10.5 to −2.6 |
| Severe | 4 (0.4) | 3.1-4.9 | 5 (0.3) | 4.3-5.7 | 11.1 (0.8) | 9.4-12.7 | −34.1 (2.8) | −39.7 to −28.6 | −20.2 (2) | −24.2 to −16 |
| Anemia | −0.2 (0.4) | −0.9 to 0.5 | 1.40 (0.3) | 0.8-2.1 | 5 (0.7) | 3.6-6.4 | −18.3 (2.4) | −23.1 to −13.5 | −2.7 (1.7) | −6.2 to 0.6 |
| Heart failure | 0.3 (0.6) | −0.9 to 1.5 | 1.99 (0.6) | 0.9-3.1 | 5.5 (1.2) | 3.1-7.9 | −32.4 (4.1) | −40.5 to −24.3 | −2 (3) | −7.9 to 3.8 |
| Pulmonary hypertension | 0.4 (0.6) | −0.7 to 1.5 | 1.53 (0.5) | 0.5-2.6 | 4.4 (1.1) | 2.1-6.6 | −21 (3.8) | −28.5 to −13.3 | −7.1 (2.7) | −12.4 to −1.9 |
| Liver failure | 2.7 (1.3) | 0.2-5.1 | 3.1 (1.1) | 0.92-5.32 | 8.7 (2.5) | 3.9-13.6 | −30 (8.5) | −46.6 to −13.2 | −6.8 (5.8) | −18.3 to 4.7 |
| Nosebleed treatment | 0.2 (0.6) | −0.9 to 1.3 | 1.6 (0.5) | 0.60-2.60 | 4.9 (1.1) | 2.7-7 | −22.6 (3.8) | −30.1 to −15.2 | −1.6 (2.6) | −6.8 to 3.6 |
| Shortness of breath | 1.7 (0.4) | 0.9-2.5 | 2.6 (0.3) | 1.99-3.31 | 8.3 (0.7) | 6.9-9.7 | −25 (2.5) | −29.9 to −20 | −7.9 (1.8) | −11.5 to −4.3 |
| Hemoptysis | 1.3 (0.4) | 0.5-2 | 1.6 (0.3) | 0.96-2.29 | 4.1 (0.7) | 2.6-5.5 | −12.5 (2.5) | −17.5 to −7.4 | −5.9 (1.8) | −9.4 to −2.5 |
| Headache | 2 (0.4) | 1.3-2.8 | 2.1 (0.3) | 1.46-2.77 | 5.9 (0.7) | 4.6-7.4 | −17.6 (2.5) | −22.5 to −12.6 | −7.4 (1.7) | −10.9 to −3.9 |
| Seizure | 2.9 (0.7) | 1.5-4.4 | 3.06 (0.6) | 1.81-4.32 | 4.3 (1.4) | 1.5-7.2 | −12.5 (5) | −22.2 to −2.8 | −4.5 (3.4) | −11.3 to 2.1 |
| Stroke | 0.3 (0.6) | −0.8 to 1.5 | −0.07 (0.5) | −1.09 to 0.94 | 1.2 (1.2) | −1.1 to 3.5 | −11.4 (4) | −19.2 to 3.4 | −0.6 (2.7) | −6.1 to 4.7 |
| Edema | 0.8 (0.4) | 0.04-1.6 | 1.66 (0.3) | 0.99-2.34 | 4.6 (1.1) | 3.1-6.1 | −18.6 (2.6) | −23.6 to −13.5 | −4.7 (1.8) | −8.2 to −1.1 |
| Genetic mutation | 0.4 (0.4) | −0.4 to 1.1 | −0.1 (0.3) | −0.8 to 0.4 | 0.1 (0.6) | −1.1 to 1.3 | -0.9 (2.2) | −5.4 to 3.4 | 0.5 (1.7) | −2.6 to 3.7 |

HHT, hereditary hemorrhagic telangiectasia; SD, standard deviation.

**eTable 2. Tukey’s Honestly Significant Difference (Tukey’s HSD) Test Analysis Results**

| **Variable** | **Mean NOSE-HHT** | **Mean ESS** | ***P* (ANOVA)** |
| --- | --- | --- | --- |
| Overall | 1.6 (0.8) | 5.0 (2.1) |  |
| HADS-A categories |  |  | <.001 |
| Normal (0-7) | 1.3 (0.7) | 4.5 (1.9) |  |
| Borderline abnormal (8-10) | 1.7 (0.8) | 5.2 (2.2) |  |
| Abnormal (11-21) | 2.1 (0.8) | 5.7 (2.2) |  |
| HADS-D categories |  |  | <.001 |
| Normal (0-7) | 1.3 (0.7) | 4.6 (2.0) |  |
| Borderline abnormal (8-10) | 1.9 (0.8) | 5.3 (2.0) |  |
| Abnormal (11-21) | 2.4 (0.7) | 6.5 (2.2) |  |

Abbreviations: ANOVA, analysis of variance; ESS, Epistaxis Severity Score; HADS-A, Hospital Anxiety and Depression Scale (Anxiety); HADS-D: Hospital Anxiety and Depression Scale (Depression); NOSE-HHT, Nasal Outcome Score for Epistaxis in Hereditary Hemorrhagic Telangiectasia.

**eTable 3. Kruskal-Wallis Test**

| **Variable** | **ESS** | ***P* value** | **Nose-HHT** | ***P* value** | **HADS-D** | ***P* value** | **HADS-A** | ***P* value** |
| --- | --- | --- | --- | --- | --- | --- | --- | --- |
| GI AVMs | 5.4 | .039 | 1.8 | .148 | 6 | .617 | 7 | .017 |
| Liver AVMs | 5.3 | .132 | 1.7 | .565 | 5 | .449 | 7 | .009 |
| Lung AVMs | 4.6 | .001 | 1.4 | .001 | 6 | .421 | 8 | .355 |
| Bleeding from GI AVMs | 5.4 | .054 | 1.8 | .041 | 6 | .112 | 7 | .194 |
| Genetic mutation associated with HHT | 5.2 | .094 | 1.8 | .181 | 6 | .398 | 7 | .137 |
| Another mutation/not tested/not known | 5.3 | .094 | 1.8 | .181 | 6 | .398 | 7 | .137 |
| Eng/HHT1 | 4.3 | .094 | 1.4 | .181 | 6 | .398 | 8 | .137 |
| Alk-1/HHT2/ACVRL | 4.4 | .094 | 1.5 | .181 | 5 | .398 | 8 | .137 |
| SMAD4 | 4.5 | .001 | 1.3 | .005 | 6 | .070 | 8 | .297 |
| Anemia | 5.6 | <.001 | 1.9 | <.001 | 6 | <.001 | 8 | .541 |
| Heart failure | 5.9 | .001 | 2.2 | <.001 | 8 | .001 | 8 | .710 |
| Pulmonary hypertension | 6.1 | <.001 | 2.2 | <.001 | 6.5 | .008 | 8 | .395 |
| Liver failure | 6.2 | .173 | 2.4 | .014 | 8 | .023 | 11 | .035 |
| Prior nosebleed related treatment | 5.2 | <.001 | 1.8 | <.001 | 6 | .002 | 8 | .810 |
| Shortness of breath | 5.5 | <.001 | 1.9 | <.001 | 7 | <.001 | 8 | <.001 |
| Hemoptysis | 5.8 | <.001 | 2.0 | <.001 | 7 | <.001 | 9 | <.001 |
| Headache | 5.4 | <.001 | 1.9 | <.001 | 7 | <.001 | 9 | <.001 |
| Seizure | 6.2 | .004 | 2.4 | <.001 | 10 | <.001 | 10 | <.001 |

Abbreviations: AVM, arteriovenous malformation; ESS, Epistaxis Severity Score; GI, gastrointestinal; HADS-A, Hospital Anxiety and Depression Scale (Anxiety); HADS-D: Hospital Anxiety and Depression Scale (Depression); NOSE-HHT, Nasal Outcome Score for Epistaxis in Hereditary Hemorrhagic Telangiectasia.

e**Table 4. Symptoms for Which the Participant Would Like to See Improvement With Treatment** (Answer: “Yes, I would like to see this symptom improved with treatment.”)

| **Symptom** | **n (%)** |
| --- | --- |
| Nosebleeds | 521 (92.2) |
| Shortness of breath | 344 (60.9) |
| Exercise intolerance | 333 (58.9) |
| Fatigue | 446 (78.9) |
| Coughing up blood | 150 (26.5) |
| Vomiting red blood or dark clots | 90 (15.9) |
| Red blood or dark blood in stool | 155 (27.4) |
| Headaches | 280 (49.6) |
| Seizures | 45 (8) |
| Legs or feet swelling | 176 (31.2) |
| Numbness | 149 (26.4) |
| Other symptoms | 51 (9) |

**eTable 5. Five-point Likert scale of Importance for Patients to Improve HHT-Related Diagnoses**

| **Category** | **n (%)** |
| --- | --- |
| Anemia |  |
| Not at all important | 0 |
| Slightly important | 8 (1.4) |
| Somewhat important | 17 (3) |
| Moderately important | 59 (10.4) |
| Extremely important | 268 (47.4) |
| Heart Failure |  |
| Not at all important | 0 |
| Slightly important | 0 |
| Somewhat important | 1 (0.2) |
| Moderately important | 5 (0.9) |
| Extremely important | 43 (7.6) |
| Pulmonary Hypertension |  |
| Not at all important | 0 |
| Slightly important | 1 (0.2) |
| Somewhat important | 7 (1.2) |
| Moderately important | 7 (1.2) |
| Extremely important | 47 (8.3) |
| Liver Failure |  |
| Not at all important | 1 (0.2) |
| Slightly important | 0 |
| Somewhat important | 0 |
| Moderately important | 2 (0.4) |
| Extremely important | 9 (1.6) |
| Other HHT related diagnoses |  |
| Yes | 252 (44.6) |
| No | 285 (50.4) |

HHT, hereditary hemorrhagic telangiectasia

**eTable 6. Five-point Likert Scale of Degree of HHT Impact in Areas of Functioning**

| \| **Area of Functioning** \| **n (%)** \| \| --- \| --- \| \| Physical functioning \|  \| \| Not at all impacted \| 73 (12.9) \| \| Slightly impacted \| 115 (20.4) \| \| Somewhat impacted \| 95 (16.8) \| \| Moderately impacted \| 135 (23.9) \| \| Extremely impacted \| 143 (25.3) \| \| Cognitive functioning \|  \| \| Not at all impacted \| 186 (32.9) \| \| Slightly impacted \| 112 (19.8) \| \| Somewhat impacted \| 94 (16.6) \| \| Moderately impacted \| 102 (18.1) \| \| Extremely impacted \| 64 (11.3) \| \| Emotional functioning \|  \| \| Not at all impacted \| 77 (13.6) \| \| Slightly impacted \| 131 (23.2) \| \| Somewhat impacted \| 105 (18.6) \| \| Moderately impacted \| 125 (22.1) \| \| Extremely impacted \| 124 (21.9) \| \| Social functioning \|  \| \| Not at all impacted \| 120 (21.2) \| \| Slightly impacted \| 109 (19.3) \| \| Somewhat impacted \| 114 (20.2) \| \| Moderately impacted \| 114 (20.2) \| \| Extremely impacted \| 104 (18.4) \| \| Role functioning \|  \| \| Not at all impacted \| 112 (19.8) \| \| Slightly impacted \| 101 (17.9) \| \| Somewhat impacted \| 102 (18.1) \| \| Moderately impacted \| 105 (18.6) \| \| Extremely impacted \| 140 (24.8) \|     **eTable 7. Five-Point Likert Scale of Importance for Patients to Improve Different Areas of Functioning**   \| **Area of Functioning** \| **n (%)** \| \| --- \| --- \| \| Physical functioning \|  \| \| Not important at all \| 50 (8.8) \| \| Slightly important \| 40 (7.1) \| \| Somewhat important \| 73 (12.9) \| \| Moderately important \| 108 (19.1) \| \| Extremely important \| 289 (51.2) \| \| Cognitive functioning \|  \| \| Not important at all \| 99 (17.5) \| \| Slightly important \| 38 (6.7) \| \| Somewhat important \| 61 (10.8) \| \| Moderately important \| 86 (15.2) \| \| Extremely important \| 266 (47.1) \| \| Emotional functioning \|  \| \| Not important at all \| 56 (9.9) \| \| Slightly important \| 61 (10.8) \| \| Somewhat important \| 75 (13.3) \| \| Moderately important \| 111 (19.6) \| \| Extremely important \| 253 (44.8) \| \| Social functioning \|  \| \| Not important at all \| 76 (13.5) \| \| Slightly important \| 60 (10.6) \| \| Somewhat important \| 69 (12.2) \| \| Moderately important \| 118 (20.9) \| \| Extremely important \| 233 (41.2) \| \| Role functioning \|  \| \| Not important at all \| 76 (13.5) \| \| Slightly important \| 45 (8.0) \| \| Somewhat important \| 62 (11.0) \| \| Moderately important \| 128 (22.7) \| \| Extremely important \| 243 (43.0) \| |
| --- | --- | --- | --- | --- | --- | --- | --- | --- | --- | --- | --- | --- | --- | --- | --- | --- | --- | --- | --- | --- | --- | --- | --- | --- | --- | --- | --- | --- | --- | --- | --- | --- | --- | --- | --- | --- | --- | --- | --- | --- | --- | --- | --- | --- | --- | --- | --- | --- | --- | --- | --- | --- | --- | --- | --- | --- | --- | --- | --- | --- | --- | --- | --- | --- | --- | --- | --- | --- | --- | --- | --- | --- | --- | --- | --- | --- | --- | --- | --- | --- | --- | --- | --- | --- | --- | --- | --- | --- | --- | --- | --- | --- | --- | --- | --- | --- | --- | --- | --- | --- | --- | --- | --- | --- | --- | --- | --- | --- | --- | --- | --- | --- | --- | --- | --- | --- | --- | --- | --- | --- | --- | --- | --- | --- |
