# Supplementary material for: Quantifying the burden of hereditary hemorrhagic telangiectasia on quality of life and psychological health: a cross-sectional study
Source: Orphanet J Rare Dis. 2025 Mar 7;20:109. doi: 10.1186/s13023-025-03620-8 (PMC11889918; doi:10.1186/s13023-025-03620-8)
Supplement: Supplementary file 3 — Supplementary Material 3 [file 13023_2025_3620_MOESM3_ESM.docx]

Orphanet J Rare Dis Authorship Requirements (must initial all 3 boxes)

|  | Author Initials |
| --- | --- |
| I have made substantial contributions to conception OR design of the work; OR the acquisition, analysis, OR interpretation of data; OR the creation of new software used in the work; OR have drafted the work or substantively revised it | AJG, MLB, EEL, MRT, YZ, JM, PG, TG, CRW |
| I have approved the submitted version (and any substantially modified version that involves the author's contribution to the study); | AJG, MLB, EEL, MRT, YZ, JM, PG, TG, CRW |
| I have agreed both to be personally accountable for the author's own contributions and to ensure that questions related to the accuracy or integrity of any part of the work, even ones in which the author was not personally involved, are appropriately investigated, resolved, and the resolution documented in the literature. | AJG, MLB, EEL, MRT, YZ, JM, PG, TG, CRW |
