# Supplementary material for: Quantifying the burden of hereditary hemorrhagic telangiectasia on quality of life and psychological health: a cross-sectional study
Source: Orphanet J Rare Dis. 2025 Mar 7;20:109. doi: 10.1186/s13023-025-03620-8 (PMC11889918; doi:10.1186/s13023-025-03620-8)
Supplement: Supplementary file 4 — Supplementary Material 4 [file 13023_2025_3620_MOESM4_ESM.docx]

[Orphanet J Rare Dis] May 29, 2024

**Quantifying the burden of hereditary hemorrhagic telangiectasia on quality of life and psychological health: A cross-sectional study**

Anna J. Gong, MD,^1^ Marisabel Linares Bolsegui, MD, MPH,^1^ Emerson E. Lee, MD,^1^ Matthew R. Tan, MD,^1^ Yong Zeng, MD, ScM,^2^ Jianqiao Ma, ScM,^2^ Prateek Gowda, MD,^1^ Tushar Garg, MD,^1^ Clifford R. Weiss, MD^1^

^1^Department of Radiology and Radiological Science, The Johns Hopkins School of Medicine, Baltimore, MD.

^2^Biostatistics, Epidemiology, and Data Management (BEAD) Core. Department of Pediatrics, Johns Hopkins University School of Medicine, Baltimore, MD

**Corresponding Author:** Clifford R. Weiss, MD, Professor of Radiology, The Johns Hopkins University School of Medicine, 7203 Sheikh Zayed Tower, Suite 7, 1800 Orleans Street, Baltimore, Maryland 21287, USA, Email: cweiss@jhmi.edu

**Counts**: Manuscript word count: 3905

**Quantifying the burden of hereditary hemorrhagic telangiectasia on quality of life and psychological health: A cross-sectional study**

**Abstract**

**Background:** Despite the considerable burden that hereditary hemorrhagic telangiectasia (HHT) imposes, few studies have investigated its effect on health-related quality of life (HRQoL). We aimed to assess the impact of HHT on psychosocial QoL and identify demographic and clinical factors associated with lower QoL.

**Methods:** We conducted an international, cross-sectional study of 1042 adults with HHT within the Cure HHT network, between 2022 and 2023. We used an online survey that included 5 standardized instruments to evaluate patients’ perceptions of the impact of HHT on their QoL: Epistaxis Severity Score (ESS); Nasal Outcome Score for Epistaxis in Hereditary Hemorrhagic Telangiectasia (NOSE-HHT); Patient-Reported Outcomes Measurement Information System (PROMIS) Fatigue – Short Form 8a – Fatigue interfere scale (PROMIS-Fatigue 8a); Hospital Anxiety and Depression Scale (HADS-A and HADS-D); and Short Form Health Survey (SF-36). Statistical analyses included Spearman’s correlations, univariate analyses, Tukey’s honestly significant difference, and Kruskal-Wallis tests.

**Results:** 565/1042 (54%) participants completed the survey. The most common symptoms were epistaxis 521/565 (92%) and fatigue 446/565 (79%). There were strong positive correlations between HADS-A and ESS (2.6 [95% CI 1.7-3.6]) and NOSE-HHT (4 [3.2-5]); HADS-D and ESS (1.4 [1.3-1.5]) and NOSE-HHT (4.4 [3.4-5.7]); PROMIS Fatigue 8a and ESS (8.2 [6.3-10]) and NOSE-HHT (5.9 [5.2-6.6]); and SF-36 scores and ESS (−26.4 [−33 to −19.9]) and NOSE-HHT (−33.1 [−39.7 to −28.6]). Liver failure and seizures indicated a higher likelihood of depression (3.1 [1-5.2]), anxiety (3 [0.6-5.4]), and fatigue (9.6 [4.7-14.5]). Seizures were associated with depression (2.9 [1.8-3.9]), anxiety (2.9 [1.7-4.1]), and fatigue (5 [2.34-7.7]). Participants expressed a substantial effect on their physical (143/560 [25%]), role (140/556 [25%]), emotional (124/554 [22%]), social (104/556 [18%]), and cognitive (64/550 [11%]) functioning. However, more participants considered extremely important to improve their physical (289/560 [51%]), cognitive (266/550 [47%]), role (253/556 [43%]), emotional (243/554 [45%]), and social (233/556 [41%]) functioning affected by HHT.

**Conclusions:** Severe epistaxis is associated with higher rates of depression, anxiety, and fatigue. Participants expressed desire for improvement in a broad range of functional domains disturbed by HHT. This suggests a need for increased awareness, resources, and more effective interventions to improve the QOL of patients with HHT.

**Keywords:** HHT, Quality of life (QOL), Patient-Reported Outcome (PRO)

**Background**

Hereditary hemorrhagic telangiectasia (HHT) is an autosomal dominant multisystem vascular disorder, characterized by the presence of arteriovenous malformations (AVMs) and telangiectasias, especially affecting the skin, nose, lungs, liver, gastrointestinal tract, and central nervous system [1]. The clinical spectrum of this condition is broad, encompassing symptoms ranging from mild to severe epistaxis that frequently result in significant anemia requiring ongoing blood transfusions [1–3]. Gastrointestinal (GI) tract involvement is common. GI AVMs affect up to 80% of patients, with about 30% experiencing bleeding, a major contributor to the anemia seen in nearly half of these patients [1,2]. Seventy percent of the HHT population experiences liver AVMs, leading to complications such as high-output heart failure, portal hypertension, biliary disease, and neurologic dysfunction [1,4]. Furthermore, lung AVMs, which are found in up to 40% of individuals, may cause symptoms like dyspnea, pulmonary hypertension, hemorrhage, septic emboli, and stroke. Additionally, cerebral AVMs affect 10% to 15% of patients, heightening the likelihood of neurologic complications including migraines, seizures, and brain abscesses [5]. This variability in clinical presentation not only complicates diagnosis and management, but also considerably affects the quality of life (QoL) of individuals with HHT [6].

Health-related QoL (HRQoL) is an essential indicator to assess the broader impact of chronic conditions like HHT, beyond the immediate physical symptoms, to quantify the influence of a medical condition, and to evaluate the effectiveness of therapeutic interventions. It encompasses the patient's perception of their position in life, in the context of culture, value systems, goals, expectations, standards, and concerns [7–9]. Hence, improvements in HRQoL are recognized as a primary outcome and a critical determinant of therapeutic interventions [10]. Despite the known extensive burden of HHT, research into its psychosocial outcomes remains sparse. Studies have explored the prevalence of anxiety and depression among individuals with HHT [11–14]; however, a deep understanding of how HHT-specific manifestations affect psychosocial aspects of HRQoL requires further exploration.

Furthermore, while the physical effects of HHT, such as epistaxis and AVM-related complications, are well documented, their correlation with HRQoL, including the psychosocial dimension, and different domains of functioning have not been thoroughly investigated. The complexity of HHT symptoms and the way they are managed can profoundly affect individuals' mental health, social interactions, ability to function, and overall QoL, suggesting a substantial but underexplored area of influence on HRQoL.

This internet-based cross-sectional study aimed to investigate the impact of HHT on HRQoL in a diverse population across North America, South America, Europe, Asia, Australia, and South Africa by exploring the symptomatic burden of HHT and identifying demographic variables that could affect HRQoL. The findings are expected to inform clinical practice and future research, with the ultimate goal of improving quality of life for individuals with HHT.

**Methods**

**Study Design**

We conducted a cross-sectional survey study funded by the Cure Hereditary Hemorrhagic Telangiectasia (Cure HHT) Foundation in North America, South America, Europe, Asia, Australia, and South Africa to investigate the effects of HHT on psychosocial QoL and explore demographic and clinical factors associated with lower HRQoL.

**Study Population**

After institutional review board approval, patients were recruited from March 2022 through May 2022 through the Cure HHT network, an international patient advocacy group. An online survey link was sent via (1) the Cure HHT patient email list, (2) recruitment during a Cure HHT patient conference, or (3) the Cure HHT website. The eligibility criteria were age of 18 years or older; a definitive diagnosis of HHT, requiring at least 3 of the 4 Curaçao Criteria or positive genetic testing for any of the known HHT mutations (ENGL, ALK-1, SMAD4, HHT3/4) [14,15], and the ability to understand and participate in the survey. All participants provided written informed consent and all responses were anonymized.

**Survey Description**

Participants responded to a survey battery that included demographic information, clinical history, and questions regarding their perception of the influence of their illness experience on their QoL, the presence of different HHT-related manifestations, and 5 standardized surveys: Short Form-36 (SF-36), Hospital Anxiety Depression Scale (HADS), Epistaxis Severity Score (ESS), Nasal Outcome Score for Epistaxis in Hereditary Hemorrhagic Telangiectasia (NOSE-HHT), and Patient-Reported Outcomes Measurement Information System (PROMIS) Fatigue - Short Form 8a - Fatigue interfere scale (PROMIS-Fatigue 8a) [16].

HHT-related conditions (anemia, heart failure, pulmonary hypertension, and liver failure) were captured using dichotomous ("Yes"/"No") responses, and their extent was categorized from zero to more than four conditions. Participants were asked about their motivation for improving these diagnoses, using a 5-point Likert scale ranging from “not important at all” to “extremely important” to improve. Additionally, participants rated how various HHT-related manifestations influenced their HRQoL using Likert scales, initially indicating the extent to which they desired improvement in different HHT-related symptoms (epistaxis, shortness of breath, exercise intolerance, fatigue, hemoptysis, hematemesis, etc.). Responses were categorized as desire for improvement, no interest in treatment, or not applicable because of the absence of symptoms. Thereafter, the impact of different HHT-related manifestations on 5 functional domains (physical, cognitive, emotional, social, and role functioning) was evaluated using a 5-point adjective-rating scale ranging from “not impacted at all” to “extremely impacted.” Afterward, participants rated the importance of improving the aforementioned domains of functioning using a 5-point adjective rating scale ranging from “not important at all” to “extremely important” to improve.

**Description of the Patient-Reported Outcome Measure (PROM)s**

The Short Form-36 (SF-36) is a standardized instrument that evaluates Health-Related Quality of Life through eight QoL domains: physical functioning (PF), role physical (RP), bodily pain (BP), general health (GH), vitality (VT), social functioning (SF), role emotional (RE), and mental health (MH) [17]. In six of the eight domains, patients are asked to rate their responses on 3- to 6-point scales. The SF-36 assesses two distinct magnitudes: the Physical Component Summary (PCS), which represents the physical dimension, and the Mental Component Summary (MCS), which represents the mental dimension. Each domain contributes in different proportions to the scores of both PCS and MCS. Furthermore, the precise calculation of the PCS and MCS is dependent on specific algorithms that are coded, summed, and transformed onto a scale from 0 (worst health) to 100 (best health) [18].

The Epistaxis Severity Score (ESS), the first standardized severity scoring system in HHT (2010), is a partially validated survey that evaluates individual disease severity and treatment efficacy for HHT-related epistaxis [19,20]. It assesses six independent predictors of self-described epistaxis severity: frequency, duration, severity, anemia, blood transfusions, and the need for medical attention. After applying standardized coefficients to the responses, the sum yields a raw ESS score, which is then normalized to a 0-10 scale from no epistaxis to most severe epistaxis, with a minimal clinically important difference (MCID) of 0.71 [20,21].

The Nasal Outcome Score for Epistaxis in Hereditary Hemorrhagic Telangiectasia (NOSE-HHT) is a 29-item HHT symptom-specific survey (2020) that evaluates QOL of individuals with HHT. It assesses the effect of epistaxis severity with sensitivity to change on specific psychosocial metrics, such as physical, functional, and emotional aspects (MCID: 0.46). The result is calculated by dividing the sum of each item response by the total number of questions, obtaining a discrete score that ranges continuously from 0 to 4 [21].

The Patient-Reported Outcomes Measurement Information System (PROMIS) Fatigue - Short Form 8a - Fatigue interfere scale (PROMIS-Fatigue 8a) instrument is an assessment developed by the National Institutes of Health (NIH) to measure symptoms and quality-of-life indicators related to different chronic conditions such as multiple sclerosis and rheumatoid arthritis. It utilizes a T-score system normalized to a mean of 50 and a standard deviation (SD) of 10 based on the US general population, where higher scores indicate increased fatigue [8,22–24].

Lastly, the Hospital Anxiety Depression Scale (HADS) is a self-rated dual 14-item scale on a 4-point scoring system (from 0 to 3) that measures symptoms of anxiety (HADS-A) and depression subscore (HADS-D) during the previous 7 days. For each component the total maximum score is 21, and higher scores correspond with greater severity of the conditions. A score of 11 or above on either subscale suggests a diagnosis of anxiety or depression [25,26].

**Statistical Analysis**

Pearson’s and Spearman’s rho correlations evaluated the strength of association between the severity of epistaxis (as defined by ESS and NOSE-HHT scores) and variables such as anxiety, depression, fatigue, physical and mental functioning (assessed by HADS, PROMIS-Fatigue 8a, and SF-36). Additionally, we explored the relationship between demographic variables and the burden of HHT-related health conditions.

Multivariable linear regression was employed to determine the independent effect of epistaxis severity and different demographic factors (age, sex, race, and ethnicity) on HRQoL, while adjusting for demographic covariates and the presence of known HHT-related genetic mutations.

The association between the perceived impact of HHT and the importance attributed by participants to improving five domains of functioning (physical, cognitive, emotional, social, and role) was characterized using Likert scales and Spearman correlation analysis. Tukey’s honest significant difference (Tukey’s HSD) test was applied to compare HADS-A and HADS-D scores, as well as NOSE-HHT and ESS. The non-parametric Kruskal-Wallis test assessed mean differences in demographic and clinical manifestations related to HHT and all included PROs.

Statistical analyses were conducted using Stata Statistical Software: Release 17 (Stata Corp LLC; College Station, TX) and R software [27]. Results were reported with effect size measures to delineate the magnitude of the difference or the strength of the association between the compared groups, with a significance threshold set at *P* < .05. and 95% confidence interval (CI) defining the accuracy of the estimate. GraphPad Prism version 9.5.1 for Windows (GraphPad Software, Boston, MA) [28] was used to create figures.

**Results**

**Patient-Reported Illness Characteristics**

Of 1042 patients initially recruited, 565 (54.2%) completed the survey. The mean (standard deviation [SD]) age was 56.1 (13.6) years. Most participants were female (72.6% [410/565]), White (93.6% [529/565]), non-Hispanic (91.3% [516/565]), and from North America (57.5% [325/565]), and a majority reported having a known HHT-related genetic mutation (66.5% [376/565]) (Table 1). Anxiety was present in 22% of the participants (162/565), and depression and severe fatigue in 10% (58/565) respectively. The most affected component of the SF-36 was energy (mean score 35, SD 20.7), followed by the physical component summary (mean 40.8, SD 41.8) (Table 2). More than half the participants (51.6% [292/565]) reported having 3 or more HHT-related conditions. The mean age demonstrated a positive trend with the number of HHT-related health conditions, indicating that an increased burden of disease could be correlated with advancing age. Specifically, the mean age rose incrementally from 52.1 years in participants with no conditions to 58 years in those with four or more conditions. Notably 80.9% (72/89) of those with more than four conditions were female, pointing to a potential sex-related predisposition to developing a greater number of HHT-related health complications (Table 3).

**Correlates for Anxiety, Depression, and Fatigue in Patients with HHT**

Spearman's correlation analyses revealed a positive correlation between the severity of epistaxis and measures of anxiety, depression, and fatigue (Figure 1). The median scores for each PRO increased in proportion to the intensity of epistaxis (severe epistaxis ESS >7), evidenced by higher HADS-A (10; 95% confidence interval [CI] 7-13), HADS-D (8; 95% CI 5-11), and PROMIS-8a Fatigue scale (65.3; 95% CI 61.3-69.8) scores, respectively. Physical and mental abilities declined as the severity of the epistaxis increased. The physical component summary (PCS) of the SF-36 was substantially more affected than the mental component summary (MCS) (45 versus 56 in participants who reported severe epistaxis) (Table 4).

The multivariable linear regression analysis revealed that in patients with severe epistaxis, for each ESS unit increase, we observed an average increase of 2.4 points in HADS-A, 3.3 points in HADS-D, and 8 points in the PROMIS-Fatigue 8a, after adjusting for other factors. In addition, liver failure and shortness of breath were strongly positively correlated with higher HADS-A, HADS-D, and PROMIS-Fatigue 8a scores. Conversely, White race was negatively correlated with the NOSE-HHT score (−0.28;−0.55,−0.01) and older participants exhibited lower HADS and PROMIS-Fatigue 8a scores. These findings suggest that individuals with severe epistaxis and HHT-related comorbid diagnoses such as liver failure, seizures, and shortness of breath are more likely to experience greater anxiety, depression, and fatigue. Patients with heart failure showed the most substantial reduction in physical ability. Liver failure was the second most important covariate for a lower PCS score (ß = −30; −46.6 to −13.2). Female and elderly participants demonstrated a more pronounced negative impact on their physical ability, with lower PCS scores, while White race was associated with a higher PCS score. Age inversely correlated with all quality of life measures except the MCS, where it showed a slight positive association. No significant correlation was observed between race or sex and MCS score (Supplement 2:eTable 1). Moreover, ANOVA with Tukey’s post hoc analysis indicated that individuals categorized as abnormal HADS-A (mean [SD] 2.1 [0.8]) and HADS-D (2.4 [0.7]) had higher mean NOSE-HHT and ESS than those in the normal category (1.3 [0.7] and 4.6 [2.0], respectively) (Supplement 2:eTable 2).

**Correlates for Epistaxis Severity and Other HHT-Related Symptoms**

The Kruskal-Wallis test revealed a significant difference between the ESS categories among participants with prior epistaxis-related treatment (5.2), anemia (5.6), pulmonary hypertension (6.1), shortness of breath (5.5), hemoptysis (5.8), headache (5.4) (all p <.001); lung AVMs (4.6), SMAD4 genetic mutation (5.2), heart failure (5.9) (all p=.001); and seizures (6.2) (p=.004) (Supplement 2: eTable 3).

**Patient-reported impact and desire to improve HHT-related manifestations and areas of functioning**

The most common symptoms that affected HRQoL and prompted a strong desire for improvement were nosebleeds (92.2%), fatigue (78.9%), shortness of breath (60.9%), exercise intolerance (58.9%), and headaches (49.6%) (Supplement 2:eTable 4). Notably, anemia was rated by 47.4% of the participants as extremely important to improve (Supplement 2:eTable 5).

The perceived impact and importance of improving different HHT manifestations on separate areas of functioning provided noteworthy insights into the wide-ranging consequences of HHT on patients’ daily lives (Figure 2). Participants reported their physical functioning as the most “extremely impacted” domain (25.3%), followed by role functioning (24.8%), emotional functioning (21.9%), social functioning (18.4%), and cognitive functioning (11.3%). Nearly half of the participants (49.3%) reported either “extremely” or “moderately” impacted physical functioning (Supplement 2:eTable 6). Moreover, the proportion of participants who reported it as “extremely important” to improve their physical, cognitive, and emotional functioning was approximately twice as great as that of participants reporting these domains as “extremely impacted” (Supplement 2:eTable 7). Participants who rated their cognitive (17.9%) and physical (14.1%) domains as “somewhat or slightly impacted” still considered it “extremely important” to improve these areas of functioning. Spearman correlation analysis revealed a significant positive correlation between the 5-point adjective rating scales measuring the level of impact and the level of importance of improving all 5 domains of functioning: emotional functioning (r=0.64), role functioning (r=0.64), social functioning (r=0.62), physical functioning (r = 0.59) (all p <.0001), and cognitive functioning (r=0.63) (p <.001).

**Discussion**

Our findings provide insight into how HHT-related manifestations affect the psychosocial health of individuals living with this condition. People with HHT often suffer from more than three HHT-related conditions and confront substantial challenges in their daily lives, resulting in a decline in their HRQoL. Symptoms contributing to this decline included epistaxis, fatigue, shortness of breath, exercise intolerance, and headaches, and these were strongly correlated with anxiety, depression, and fatigue. In addition, anemia emerged as the condition that participants prioritized for improvement. Our analysis reveals a strong correlation between epistaxis severity and lower QOL measurements, emphasizing the profound psychosocial implications of this symptom.

This study underscores the heterogeneous nature of HHT and the need for comprehensive management strategies to address the diverse manifestations of the disease. Our outcomes revealed that women and older participants experienced a more negative effect on their physical well-being. The majority of study subjects identified as White, and they exhibited better physical functioning than the other racial and ethnic groups. In addition, the representation of other ethnicities was considerably lower, which may reflect the demographic composition of the cohort or suggest potential disparities in disease prevalence and health outcomes across different races within the HHT population. Age was negatively correlated with PCS but positively correlated with MCS, suggesting an association between increasing age and improved mental well-being. Despite variations in the perceived impact severity of functional limitations, participants consistently expressed a strong desire for improvement across all domains, suggesting that they recognize the benefit of addressing these specific domains, even if the perceived impact may not be as pronounced as the strong desire for improvement.

The negative outcomes of HHT are comparable to those of other chronic diseases, resulting in lower levels of physical and psychological QoL compared to those without this condition [8,12,13,16,20]. Various HHT-related conditions have consistently been linked to poorer outcomes and reduced QoL [6,13,14,18,29,30]. Different standardized validated instruments, such as the ESS [14,20], SF-36 [13,29], PROMIS- Fatigue 8a [31], EuroQol 5-dimension 3-level version [32], social index [12], non-standardized questionnaires [30], and interviews [11], have been used to measure physical, emotional, and social domains in individuals with HHT. However, the influence of this condition on HRQoL is often underestimated, and the understanding of HRQoL may still benefit from further research to explore dimensions not fully addressed by existing instruments [6,31,33].

Important associations have been reported between the severity of epistaxis and fatigue levels in individuals with HHT, a condition often complicated by fatigue due to iron deficiency anemia from nosebleeds [34,35]. Nevertheless, while the relationship between general fatigue and HHT is documented [36,37], our analysis contributes to the literature by specifically examining how variations in epistaxis severity influence fatigue levels. This aspect has not been distinctly addressed previously, revealing a gap in direct data correlating epistaxis severity with fatigue levels in HHT [1,2]. Seizures, an uncommon but disturbing type of HHT-related complication, were identified as a significant predictor (p <0.05) of worse HRQoL, and the established association between epilepsy and depression and lower QoL corroborates our findings [10,38,39]. Current research on the consequences of multiple chronic conditions on HRQoL is limited [40]. Despite this, it seems plausible that there is a synergistic effect of different HHT-related manifestations, including epistaxis, liver failure, cerebral AVM, and seizures, on QoL and psychological health.

Moreover, patients with HHT and comorbid liver and gastrointestinal AVMs demonstrated remarkably lower PCS scores than the general population (43.8 vs 50). Previous studies have indicated that patients with New York Heart Association (NYHA) class III heart failure exhibit SF-36 scores only one-third as high as the non-diseased population in the domains of physical functioning, bodily pain, general health, vitality, and role functioning [41–44]. This study builds upon prior research by specifically examining the significance of heart failure within patients with HHT, reaffirming the connection between heart failure and diminished physical ability.

Our analyses revealed that demographic factors such as age and sex have an effect on the HRQoL of patients with HHT, as seen in other chronic conditions. The phenotype of HHT is age-dependent, and most of the patients exhibit a complete penetrance after the age of 40 (e.g., GI bleeding is rare before age 50 years) [1,45]. Likewise, advanced age in patients with HHT has been previously associated with increased physical limitations and worse psychosocial quality of life and QoL scores [12–14,46]. Our findings showed an increased number of HHT-related comorbid diagnoses in elderly participants, but we found better mental and psychological QoL in the same age group when compared to their younger counterparts. Although young people with HHT usually experience less severe symptoms and older patients bear the vast majority of the HHT-related disease burden, QoL might improve as individuals adapt to living with their illness [6,21,47]. Older adults with HHT may have developed more mechanisms to cope with their disease because of their experience navigating their diagnosis, possibly decreasing anxiety and fatigue [6]. These findings highlight the significance of age and experience in adapting to the effects of HHT on individuals’ overall QoL.

The pronounced negative consequences on physical functioning in women may reflect broader trends of sex-specific disparities in health outcomes and suggest that female patients with HHT might experience more severe physical manifestations or have different coping mechanisms compared to male patients. Previous studies have indicated that women have more pronounced liver involvement, a higher prevalence of pulmonary AVMs, and a greater requirement for invasive procedures than men [48,49]. Because of their longer lifespan, women endure a higher prevalence of physical and psychological illnesses and lower QoL than males [49,50].

Additional evidence has revealed that Asian individuals have a higher incidence of pulmonary AVMs when compared with the other racial and ethnic groups, while Hispanic or Latino individuals may develop more cerebral AVMs [51,52]. Understanding the association between race and ethnicity and psychosocial health related to HHT will provide valuable information for identifying potential health disparities to develop tailored and equitable interventions.

**Limitations**

Several limitations should be acknowledged. First, the use of self-reported survey data and reliance on a specific patient advocacy group network may introduce selection and response biases, potentially limiting the generalizability of the findings across different populations. Moreover, the cross-sectional survey design prevents us from establishing causal relationships. We must recognize that the full extent of HHT’s negative effects on patients’ HRQoL may not be entirely understood because of the absence of a dedicated HHT-specific instrument capable of accurately capturing PROs for this condition [31]. Only 2 of the 4 validated QoL instruments specific to HHT (ESS and NOSE-HHT) were included, as neither the QoL-HHT nor the HHT-QoL instrument was published at the time of this study [6,31].

**Conclusions**

This study addresses a gap in the literature by examining how HHT-related manifestations affect patients’ psychosocial health and QOL. While severe epistaxis has predominated as one of the most prevalent and debilitating symptoms, it provides only a narrow view of the impact of HHT. Our findings emphasize the multifaceted impact of HHT on patients' lives and how several HHT-related manifestations might be associated with adverse psychological outcomes and low QoL. Also, various demographic factors such as age, sex, and race have shown notable correlations with reduced physical ability and adverse psychological outcomes. Acknowledging the full scope of HHT's negative effects is essential for a more nuanced understanding and management of this condition, improving patient care and effective target interventions.

**Abbreviations**

AVM arteriovenous malformation

Cure HTT Cure Hereditary Hemorrhagic Telangiectasia

ESS Epistaxis Severity Score

HADS-A Hospital Anxiety and Depression Scale – Anxiety

HADS-D Hospital Anxiety and Depression Scale – Depression

HHT hereditary hemorrhagic telangiectasia

MCS mental component summary

NOSE-HHT Nasal Outcome Score for Epistaxis in Hereditary Hemorrhagic Telangiectasia

PCS physical component summary

PRO patient-reported outcome

PROMIS Patient-Reported Outcomes Measurement Information System

QoL quality of life

SF-36 Short Form Health Survey (SF-36)

Tukey’s HSDS Tukey’s honestly significant difference

**Declarations**

- Ethics approval and consent to participate: The study was approved by the institutional review board of The Johns Hopkins Medical institutions, and all recruitment and data collection procedures were performed following the relevant ethical standards. Written informed consent has been obtained from each patient.
- Consent for publication: We confirm that all authors have approved the manuscript for publication.
- Availability of data and material: The datasets used in this study are not publicly available due to privacy and confidentiality concerns. However, interested individuals can request access to the data from the corresponding author, subject to approval by The Johns Hopkins Medical Institutions.
- Competing interests: None.
- Funding**:** This study was funded through a grant provided by the Cure HHT Foundation.
- Authors' contributions: AJG conceptualized the study, collected the data, and edited the manuscript, MLB wrote the manuscript and performed data analysis, EEL, MRT, YZ, and JM, did the data analysis, PG and TG contributed to data collection. CRW supervised the entire study, and reviewed and edited the manuscript. All authors read and approved the final manuscript.
- Acknowledgements: For editorial assistance, we thank Denise Di Salvo, MS, in the Editorial Services group of The Johns Hopkins Department of Orthopaedic Surgery.

**References**

1. McDonald J, Stevenson DA. Hereditary Hemorrhagic Telangiectasia. In: Adam MP, Everman DB, Mirzaa GM, Pagon RA, Wallace SE, Bean LJ, et al., editors. GeneReviews® [Internet]. Seattle (WA): University of Washington, Seattle; 1993 [cited 2023 Feb 12]. Available from: http://www.ncbi.nlm.nih.gov/books/NBK1351/

2. Locke T, Gollamudi J, Chen P. Hereditary Hemorrhagic Telangiectasia (HHT). StatPearls [Internet]. Treasure Island (FL): StatPearls Publishing; 2022 [cited 2023 Feb 12]. Available from: http://www.ncbi.nlm.nih.gov/books/NBK578186/

3. Bayrak-Toydemir P, Mao R, Lewin S, McDonald J. Hereditary hemorrhagic telangiectasia: An overview of diagnosis and management in the molecular era for clinicians. Genet Med [Internet]. 2004 [cited 2023 Jun 9];6:175–91. Available from: https://www.nature.com/articles/gim200439

4. Serra MM, Besada CH, Cabana Cal A, Saenz A, Stefani CV, Bauso D, et al. Central nervous system manganese induced lesions and clinical consequences in patients with hereditary hemorrhagic telangiectasia. Orphanet J Rare Dis. 2017;12:92.

5. Fulbright RK, Chaloupka JC, Putman CM, Sze GK, Merriam MM, Lee GK, et al. MR of hereditary hemorrhagic telangiectasia: prevalence and spectrum of cerebrovascular malformations. AJNR Am J Neuroradiol. 1998;19:477–84.

6. Le TTT, Martinent G, Dupuis-Girod S, Parrot A, Contis A, Riviere S, et al. Development and validation of a quality of life measurement scale specific to hereditary hemorrhagic telangiectasia: the QoL-HHT. Orphanet J Rare Dis. 2022;17:281.

7. Selim AJ, Qian SX, Rogers W, Arya D, Simmons K, Shapiro GD, et al. Health Status in Adults With Chronic Conditions: Intervention Strategies for Improving Patient-Reported Outcomes. J Ambul Care Manage. 2019;42:2–20.

8. Rothrock NE, Kaiser KA, Cella D. Developing a valid patient-reported outcome measure. Clin Pharmacol Ther. 2011;90:737–42.

9. Kotronoulas G., Maguire R., Harrow A., Di Domenico D., Croy S., Kearney N. What is the value of the routine use of patient-reported outcome measures toward improvement of patient outcomes, processes of care, and health service outcomes in cancer care? A systematic review of controlled trials. [cited 2023 Mar 14]; Available from: https://core.ac.uk/reader/158368143?utm_source=linkout

10. Megari K. Quality of life in chronic disease patients. Health Psychol Res. 2013;1:e27–e27.

11. Martinent G, Carrot M, Chirac A, Dupuis-Girod S, Fargeton A-E, Blois Da Conceição S, et al. Hereditary hemorrhagic telangiectasia and health-related quality of life: a qualitative investigation. Qual Life Res [Internet]. 2020 [cited 2023 May 26];29:1291–9. Available from: https://doi.org/10.1007/s11136-020-02415-7

12. Zarrabeitia R, Fariñas-Álvarez C, Santibáñez M, Señaris B, Fontalba A, Botella LM, et al. Quality of life in patients with hereditary haemorrhagic telangiectasia (HHT). Health Qual Life Outcomes [Internet]. 2017 [cited 2023 May 24];15:19. Available from: https://www.ncbi.nlm.nih.gov/pmc/articles/PMC5259834/

13. Geisthoff UW, Heckmann K, D’Amelio R, Grünewald S, Knöbber D, Falkai P, et al. Health-related quality of life in hereditary hemorrhagic telangiectasia. Otolaryngology - Head and Neck Surgery [Internet]. 2007 [cited 2023 May 24];136:726.e1-726.e10. Available from: https://www.sciencedirect.com/science/article/pii/S0194599806036060

14. Merlo CA, Yin LX, Hoag JB, Mitchell SE, Reh DD. The effects of epistaxis on health-related quality of life in patients with hereditary hemorrhagic telangiectasia. International Forum of Allergy & Rhinology [Internet]. 2014 [cited 2023 Feb 17];4:921–5. Available from: https://onlinelibrary.wiley.com/doi/abs/10.1002/alr.21374

15. Shovlin CL, Guttmacher AE, Buscarini E, Faughnan ME, Hyland RH, Westermann CJ, et al. Diagnostic criteria for hereditary hemorrhagic telangiectasia (Rendu-Osler-Weber syndrome). Am J Med Genet. 2000;91:66–7.

16. Cross-sectional, survey study measuring Health Related Quality of Life (HRQOL) of patients with Hereditary Hemorrhagic Telangiectasia [Internet]. CureHHT. [cited 2023 May 24]. Available from: https://curehht.org/research_project/hopkins-hht-hrqol-tool/

17. Lins L, Carvalho FM. SF-36 total score as a single measure of health-related quality of life: Scoping review. SAGE Open Medicine [Internet]. 2016 [cited 2024 Apr 18];4. Available from: https://www.ncbi.nlm.nih.gov/pmc/articles/PMC5052926/

18. Brazier JE, Harper R, Jones NM, O’Cathain A, Thomas KJ, Usherwood T, et al. Validating the SF-36 health survey questionnaire: new outcome measure for primary care. BMJ. 1992;305:160–4.

19. Gong AJ, Bolsegui ML, Lee EE, Mathai SC, Weiss CR. Assessing the Psychometric Validity of the Epistaxis Severity Score: Internal Consistency and Test–Retest Reliability. Am J Rhinol�Allergy [Internet]. 2024 [cited 2024 Mar 25];38:38–46. Available from: http://journals.sagepub.com/doi/10.1177/19458924231207137

20. Hoag JB, Terry P, Mitchell S, Reh D, Merlo CA. An epistaxis severity score for hereditary hemorrhagic telangiectasia. The Laryngoscope [Internet]. 2010 [cited 2023 Feb 3];120:838–43. Available from: https://onlinelibrary.wiley.com/doi/abs/10.1002/lary.20818

21. Peterson AM, Kallogjeri D, Spitznagel E, Chakinala MM, Schneider JS, Piccirillo JF. Development and Validation of the Nasal Outcome Score for Epistaxis in Hereditary Hemorrhagic Telangiectasia (NOSE HHT). JAMA Otolaryngol Head Neck Surg. 2020;146:999–1005.

22. Cook KF, Bamer AM, Roddey TS, Kraft GH, Kim J, Amtmann D. A PROMIS fatigue short form for use by individuals who have multiple sclerosis. Qual Life Res [Internet]. 2012 [cited 2024 Feb 8];21:1021–30. Available from: https://doi.org/10.1007/s11136-011-0011-8

23. Terwee CB, Elsman EB, Roorda LD. Towards standardization of fatigue measurement: Psychometric properties and reference values of the PROMIS Fatigue item bank in the Dutch general population. Research Methods in Medicine & Health Sciences [Internet]. 2022 [cited 2024 Feb 8];3:86–98. Available from: https://doi.org/10.1177/26320843221089628

24. Bartlett SJ, Gutierrez AK, Butanis A, Bykerk VP, Curtis JR, Ginsberg S, et al. Combining online and in-person methods to evaluate the content validity of PROMIS fatigue short forms in rheumatoid arthritis. Quality of Life Research [Internet]. 2018 [cited 2024 Feb 8];27:2443–51. Available from: https://www.jstor.org/stable/44856487

25. Bjelland I, Dahl AA, Haug TT, Neckelmann D. The validity of the Hospital Anxiety and Depression Scale. An updated literature review. J Psychosom Res. 2002;52:69–77.

26. Stern AF. The Hospital Anxiety and Depression Scale. Occupational Medicine [Internet]. 2014 [cited 2023 May 31];64:393–4. Available from: https://doi.org/10.1093/occmed/kqu024

27. R Core Team. R: A Language and Environment for Statistical Computing [Internet]. Vienna, Austria: R Foundation for Statistical Computing; 2021. Available from: https://www.R-project.org/

28. Home - GraphPad [Internet]. [cited 2023 Oct 30]. Available from: https://www.graphpad.com/

29. Pasculli G, Resta F, Guastamacchia E, Di Gennaro L, Suppressa P, Sabbà C. Health-related quality of life in a rare disease: hereditary hemorrhagic telangiectasia (HHT) or Rendu-Osler-Weber disease. Qual Life Res. 2004;13:1715–23.

30. Loaëc M, Moriniere S, Hitier M, Ferrant O, Plauchu H, Babin E. Psychosocial quality of life in hereditary haemorrhagic telangiectasia patients. Rhin [Internet]. 2011 [cited 2023 May 24];49:164–7. Available from: https://www.rhinologyjournal.com/Abstract.php?id=968

31. Kasthuri RS, Chaturvedi S, Thomas S, Vandergrift N, Bann C, Schaefer N, et al. Development and performance of a hereditary hemorrhagic telangiectasia-specific quality-of-life instrument. Blood Advances [Internet]. 2022 [cited 2023 May 24];6:4301–9. Available from: https://ashpublications.org/bloodadvances/article/6/14/4301/485436/Development-and-performance-of-a-hereditary

32. EQ-5D-3L Health Questionnaire [Internet]. EuroQol Research Foundation. [cited 2022 Jul 13]. Available from: https://euroqol.org/eq-5d-instruments/eq-5d-3l-about/

33. Pierucci P, Lenato GM, Suppressa P, Lastella P, Triggiani V, Valerio R, et al. A long diagnostic delay in patients with Hereditary Haemorrhagic Telangiectasia: a questionnaire-based retrospective study. Orphanet Journal of Rare Diseases [Internet]. 2012 [cited 2023 Jun 9];7:33. Available from: https://doi.org/10.1186/1750-1172-7-33

34. Kasthuri RS, Montifar M, Nelson J, Kim H, Lawton MT, Faughnan ME, et al. Prevalence and predictors of anemia in hereditary hemorrhagic telangiectasia. Am J Hematol. 2017;92:E591–3.

35. Patterson AJ, Brown WJ, Powers JR, Roberts DCK. Iron deficiency, general health and fatigue: results from the Australian Longitudinal Study on Women’s Health. Qual Life Res. 2000;9:491–7.

36. Hoag JB, Merlo CA. Health-related quality of life in patients with hereditary hemorrhagic telangiectasia and pulmonary arteriovenous malformations. D69 Exercise in Health and Disease [Internet]. American Thoracic Society; 2010 [cited 2023 Oct 9]. p. A6486–A6486. Available from: https://www.atsjournals.org/doi/abs/10.1164/ajrccm-conference.2010.181.1_MeetingAbstracts.A6486

37. Lennox PA, Hitchings AE, Lund VJ, Howard DJ. The SF-36 health status questionnaire in assessing patients with epistaxis secondary to hereditary hemorrhagic telangiectasia. Am J Rhinol. 2005;19:71–4.

38. Berto P. Quality of life in patients with epilepsy and impact of treatments. Pharmacoeconomics. 2002;20:1039–59.

39. Sajobi TT, Josephson CB, Sawatzky R, Wang M, Lawal O, Patten SB, et al. Quality of Life in Epilepsy: Same questions, but different meaning to different people. Epilepsia [Internet]. 2021 [cited 2023 Jul 18];62:2094–102. Available from: https://onlinelibrary.wiley.com/doi/abs/10.1111/epi.17012

40. Heyworth IT, Hazell ML, Linehan MF, Frank TL. How do common chronic conditions affect health-related quality of life? Br J Gen Pract [Internet]. 2009 [cited 2023 Oct 19];59:e353–8. Available from: https://www.ncbi.nlm.nih.gov/pmc/articles/PMC2765853/

41. Juenger J, Schellberg D, Kraemer S, Haunstetter A, Zugck C, Herzog W, et al. Health related quality of life in patients with congestive heart failure: comparison with other chronic diseases and relation to functional variables. Heart [Internet]. 2002 [cited 2023 Jul 19];87:235–41. Available from: https://www.ncbi.nlm.nih.gov/pmc/articles/PMC1767036/

42. Masaki Hayama, Yohei Maeda, Takashi Shikina, Shun Tatehara, Go Inokuchi, Jeffrey B Hoag, et al. Validation of epistaxis severity score for hereditary hemorrhagic telangiectasia in Japan | Elsevier Enhanced Reader [Internet]. [cited 2023 Feb 16]. Available from: https://reader.elsevier.com/reader/sd/pii/S0385814621002650?token=5D67C24FEE26F13FF2BC8D5A860868C938EA85EE9F223B3965F3587529DD188A3446371F09915572E889E563876E269B&originRegion=us-east-1&originCreation=20230216153349

43. Larson AM. Liver Disease in Hereditary Hemorrhagic Telangiectasia. Journal of Clinical Gastroenterology [Internet]. 2003 [cited 2023 Jun 15];36:149. Available from: https://journals.lww.com/jcge/Abstract/2003/02000/Liver_Disease_in_Hereditary_Hemorrhagic.13.aspx

44. Nagel M, Labenz C, Wörns MA, Marquardt JU, Galle PR, Schattenberg JM, et al. Impact of acute-on-chronic liver failure and decompensated liver cirrhosis on psychosocial burden and quality of life of patients and their close relatives. Health and Quality of Life Outcomes [Internet]. 2020 [cited 2023 Jul 18];18:10. Available from: https://doi.org/10.1186/s12955-019-1268-9

45. Danesino C, Cantarini C, Olivieri C. Hereditary Hemorrhagic Telangiectasia in Pediatric Age: Focus on Genetics and Diagnosis. Pediatr Rep [Internet]. 2023 [cited 2024 Apr 18];15:129–42. Available from: https://www.ncbi.nlm.nih.gov/pmc/articles/PMC9944132/

46. Geirdal AØ, Dheyauldeen S, Bachmann-Harildstad G, et al. Living with hereditary haemorrhagic telangiectasia: Coping and psychological distress – A cross-sectional study [Internet]. [cited 2023 Jun 7]. Available from: https://www.tandfonline.com/doi/epdf/10.3109/09638288.2012.690500?needAccess=true&role=button

47. Cohen JS, Biesecker BB. Quality of life in rare genetic conditions: A systematic review of the literature. American Journal of Medical Genetics Part A [Internet]. 2010 [cited 2023 Jun 7];152A:1136–56. Available from: https://onlinelibrary.wiley.com/doi/abs/10.1002/ajmg.a.33380

48. Mora-Luján JM, Iriarte A, Alba E, Sánchez-Corral MA, Cerdà P, Cruellas F, et al. Gender differences in hereditary hemorrhagic telangiectasia severity. Orphanet Journal of Rare Diseases [Internet]. 2020 [cited 2023 Jul 19];15:63. Available from: https://doi.org/10.1186/s13023-020-1337-5

49. Fodor E, Lane L, Schippers J, van der Lippe T. Gender Differences in Quality of Life. In: Bäck-Wiklund M, van der Lippe T, den Dulk L, Doorne-Huiskes A, editors. Quality of Life and Work in Europe: Theory, Practice and Policy [Internet]. London: Palgrave Macmillan UK; 2011 [cited 2023 Jun 7]. p. 149–61. Available from: https://doi.org/10.1057/9780230299443_9

50. Kaplan RM, Anderson JP, Wingard DL. Gender differences in health-related quality of life. Health Psychol. 1991;10:86–93.

51. Galiatsatos P, Wilson C, O’Brien J, Gong AJ, Angiolillo D, Johnson J, et al. A lack of race and ethnicity data in the treatment of hereditary hemorrhagic telangiectasia: a systematic review of intravenous bevacizumab efficacy. Orphanet J Rare Dis [Internet]. 2022 [cited 2023 Jun 7];17:220. Available from: https://www.ncbi.nlm.nih.gov/pmc/articles/PMC9195340/

52. Yusuf H, Rasheed A, Kim H, Conrad MB, Hetts SW. Identifying racial disparities in hereditary hemorrhagic telangiectasia. J Neurointerv Surg. 2022;neurintsurg-2022-019162.

**Figure Legends**

**Figure 1:** Spearman correlation analysis.

**Figure 2** Five-point Likert scales of perceived impact and level of importance on HHT-related functional domains
